# The reliability and validity of the Patient Health Questionnaire-9 (PHQ-9) and PHQ-2 in patients with infertility

**DOI:** 10.1186/s12978-019-0802-x

**Published:** 2019-09-09

**Authors:** Saman Maroufizadeh, Reza Omani-Samani, Amir Almasi-Hashiani, Payam Amini, Mahdi Sepidarkish

**Affiliations:** 1 School of Nursing and Midwifery, Guilan University of Medical Sciences, Rasht, Iran; 2 Department of Medical Ethics and Law, Reproductive Biomedicine Research Center, Royan Institute for Reproductive Biomedicine, ACECR, Tehran, Iran; 3 Department of Epidemiology, School of Health, Arak University of Medical Sciences, Arak, Iran; 4 Department of Biostatistics and Epidemiology, School of Public Health, Ahvaz Jundishapur University of Medical Sciences, Ahvaz, Iran; 5 Department of Biostatistics and Epidemiology, Babol University of Medical Sciences, Babol, Iran

**Keywords:** PHQ-9, PHQ-2, Depression, Reliability, Validity, Persian, Infertility

## Abstract

**Background:**

Depression in patients with infertility often goes undiagnosed and untreated. The Patient Health Questionnaire-9 (PHQ-9) and its ultra-brief version (i.e. PHQ-2) are widely used measures of depressive symptoms. These scales have not been validated in patients with infertility. The aim of the present study was to examine the reliability and validity of the PHQ-9 and PHQ-2 in patients with infertility.

**Methods:**

In this cross-sectional study, a total of 539 patients with infertility from a referral infertility clinic in Tehran, Iran completed the PHQ-9, along with other relevant scales: the WHO-five Well-being Index (WHO-5), the Hospital Anxiety and Depression Scale (HADS), and the Generalized Anxiety Disorder-7 (GAD-7). Factor structure and internal consistency of PHQ-9 were examined via confirmatory factor analysis (CFA) and Cronbach’s alpha, respectively. Convergent validity was evaluated by relationship with WHO-5, HADS and GAD-7.

**Results:**

The mean total PHQ-9 and PHQ-2 scores were 8.47 ± 6.17 and 2.42 ± 1.86, respectively, and using a cut-off value of 10 (for PHQ-9) and 3 (for PHQ-2), the prevalence of depressive symptoms was 38.6 and 43.6%, respectively. The Cronbach’s alphas for PHQ-9 and PHQ-2 were, respectively, 0.851 and 0.767, indicating good internal consistency. The CFA results confirmed the one-factor model of the PHQ-9 (χ^2^/df = 4.29; CFI = 0.98; RMSEA = 0.078 and SRMR = 0.044). Both PHQ-9 and PHQ-2 showed moderate to strong correlation with the measures of WHO-5, HADS-depression, HADS-anxiety, and the GAD-7, confirming convergent validity. In univariate analysis, female sex, long infertility duration, and unsuccessful treatment were significantly associated with depression symptoms.

**Conclusion:**

Both PHQ-9 and PHQ-2 are brief and easy to use measures of depressive symptoms with good psychometric properties that appear suitable for routine use in patients with infertility.

## Plain English summary

Infertility is a common public health problem affecting 9% of reproductive-aged couples worldwide. Depression is one of the most common mental disorders in infertile population. A variety of self-administered instruments have been developed for assessment of depression. Among these instruments, the Patient Health Questionnaire-9 (PHQ-9) and its ultra-brief version (i.e. PHQ-2) are two of the most widely used instruments for measuring depression in research and clinical settings. Despite this, the validity and reliability of the PHQ-9 and PHQ-2 for use in patients with infertility have not been established. In this study using a sample of patients with infertility (249 men and 290 women) in Tehran, Iran, we evaluated the reliability and validity of these instruments. The mean total PHQ-9 and PHQ-2 scores were 8.47 ± 6.17 and 2.42 ± 1.86, respectively, and using a cut-off value for PHQ-9 and PHQ-2, the prevalence of depressive symptoms was 38.6 and 43.6%, respectively. Both PHQ-9 and PHQ-2 showed good internal consistency reliability. The results of confirmatory factor analysis provided support for unidimensional structure of the PHQ-9. Evidence of convergent validity of the PHQ-9 and PHQ-2 was demonstrated by a pattern of correlations with the relevant measures of depression and anxiety that was in line with theoretical predictions. Our finding showed that depression was more common in women, patients with unsuccessful treatment, and patients with long duration of infertility. In summary, both PHQ-9 and PHQ-2 are reliable and valid instruments for measuring depression symptoms in patients with infertility. Furthermore, the brevity of these instruments increases its usefulness and appropriateness for research and clinical settings, especially in large-sample survey research.

## Background

Infertility is a public health problem recognized worldwide by the World Health Organization and affects approximately 9% of reproductive-age couples [1]. It has been known to cause negative psychological, social, and emotional consequences worldwide especially in developing countries like Iran [2]. Among these consequences, depression is one of the most prevalent psychiatric disorders and adversely affects quality of life and well-being [3–6]. Previous research in patients with infertility yielded prevalence between 30 and 40% [7–10] and epidemiological studies show that depression is more common in females, as well as individuals with longer infertility duration, low educational level, and unsuccessful treatment [9, 11, 12].

Depression can be assessed by several instruments such as the Hospital Anxiety and Depression Scale (HADS), the Beck Depression Inventory-II (BDI-II), the Center for Epidemiologic Studies Depression Scale (CES-D) and the Patient Health Questionnaire (PHQ-9). Among these instruments, the PHQ-9 is one of the most frequently used measures of depression in psychology research. The PHQ-9 is a short, self-administered, and positively worded questionnaire designed to measures the severity of depression over the last 2 weeks [13]. The PHQ-9 items are based on the criteria of the DSM-IV (the Diagnostic and Statistical Manual of Mental Disorders, Fourth Edition) [13]. A two-item version (i.e. PHQ-2) was also developed as an ultra-brief instrument for settings with a limited time frame (e.g., survey research or telephone interviews) [14].

Both PHQ-9 and PHQ-2 have been validated in a variety of populations, mainly in the primary care and general hospital settings as well as in general population [15–26]; however, they have not been validated in patients with infertility. Hence, this study aims to evaluate the validity and reliability of PHQ-9 and PHQ-2 among a sample of patients with infertility. Given that most previous factor analysis studies reported one-factor structure for the PHQ-9, it was hypothesized here that the PHQ-9 would be unidimensional.

## Methods

### Participants and study design

The study sample consists of patients with infertility drawn from Infertility Treatment Clinic of Royan Institute, Tehran, Iran between May and August 2017. This center is one of the largest clinics for infertility treatment in Tehran, Iran [27]. To be eligible for this study, participants had infertility problem; be 18 years of age or older; and be able to read and write in Persian. Infertility is defined as “the failure to establish a clinical pregnancy after 12 months of regular, unprotected sexual intercourse or due to an impairment of a person’s capacity to reproduce either as an individual or with his/her partner.” [28]. The questionnaires were administered participants. We collected data in the evaluation phase of treatment using the convenience sampling method. Those who were married (i.e. couple) were asked to fill out the questionnaires separately with no discussion between them. It took about 5 min to complete the instruments. In total, 539 patients with infertility agreed to participate and filled out the questionnaires completely.

### Instruments

#### Patient Health Questionnaire-9 (PHQ-9)

The PHQ-9 is a 9-item self-report questionnaire designed to measure depression [13]. This scale evaluates each of the 9 DSM-IV (the Diagnostic and Statistical Manual of Mental Disorders, Fourth Edition) criteria for major depressive disorder. The PHQ-9 asks how often respondents have been bothered by problems in the last 2 weeks. Items are rated on a 4-point Likert-type scale, ranging from 0 (not at all) to 3 (nearly every day). Total score can range from 0 to 27, with high scores meaning high depression. Based on the original validation studies, the total score can then be interpreted as suggesting no depression (0–4), mild (5–9), moderate (10–14), moderately severe (15–19), or severe (20–27). A cut off score of 10 is suggested as indicating a possible diagnosis of depressive disorder. The Persian version of PHQ-9 has been shown to have good psychometric properties in patients with major depression [29].

#### Patient Health Questionnaire-2 (PHQ-2)

The PHQ-2 is an ultra-brief self-report instrument that contains the first two items of the PHQ-9 [14]. The total PHQ-2 score can range from 0 to 6. A cut off score of 3 is suggested as indicating a possible diagnosis of depressive disorder. The Persian version of PHQ-2 has been shown to have good psychometric properties in patients with major depression [16].

#### WHO-5 well-being index (WHO-5)

The WHO-5 is a widely used self-report instrument consisting 5 items designed to measure well-being during the last 2 weeks [30, 31]. Items are rated on a 6-point Likert scale, ranging from 0 (at no the time) to 5 (all of the time). The responses are summed, and the raw scores are transformed to a score from 0 (worst well-being) to 100 (best well-being). A score of ≤50 indicates poor well-being and suggests further evaluation into possible symptoms of depression. This scale showed good internal consistency in the present study (α = 0.858).

#### Hospital anxiety and depression scale (HADS)

The HADS is a widely used self-report instrument consisting 14 items designed to measure both anxiety (HADS-A, 7 items) and depression (HADS-D, 7 items) [32]. Items are rated on a 4-point Likert scale ranging from 0 to 3. Both subscales can range from 0 to 21, with higher scores reflecting higher level of anxiety and depression. The Persian version of HADS has been validated and widely used in patients with infertility [11, 33]. This scale showed high internal consistency in the present study (α = 0.884 for HADS-A, and α = 0.783 for HADS-D).

#### The 7-item generalized anxiety disorder (GAD-7)

The GAD-7 is a 7-item self-report instrument which is widely used to assess generalized anxiety disorder during the last 2 weeks based on DSM-IV criteria [34]. Each item is scored on a 4-point Likert scale indicating symptom frequency, ranging from 0 (not at all) to 3 (nearly every day). The GAD-7 total score can range from 0 to 21, with a score of ≥10 is indicative of generalized anxiety disorder. The Persian version of GAD-7 has been shown to have good psychometric properties and widely used in the context of infertility [35, 36]. This scale showed good internal consistency in the present study (α = 0.876).

### Statistical analysis

The confirmatory factor analysis, with maximum likelihood estimation method, was carried out in order to examine the one-factor structure of the PHQ-9. The goodness-of-fit of the model was assessed by using the chi-square/degree of freedom (χ2/df), the comparative fit index (CFI), the root mean square error of approximation (RMSEA), and the standardized root mean square residual (SRMR). Model fit was interpreted as ‘acceptable’ if χ2/df < 5, CFI > 0.9, RMSEA< 0.08, and SRMR< 0.08 (for good fit: χ2/df < 2, CFI > 0.95, RMSEA< 0.06, and SRMR< 0.05) [37, 38]. Cronbach’s alpha, inter-item correlation, and corrected-item total correlation were used to evaluate the internal consistency of the scale. Convergent validity of the scales was established by examining correlations of PHQ-9 and PHQ-2 scores with other measures relevant to depression (WHO-5, HADS, and GAD-7). Relationships of PHQ-9 and PHQ-2 with demographic/infertility variables were examined using Pearson correlation coefficient, independent t test and one-way ANOVA. All statistical analyses were done with IBM SPSS Statistics for Windows, Version 22.0 (IBM Corp., Armonk, NY, USA) and Lisrel 8.80 (Scientific Software International, Inc., Lincolnwood, IL, USA).

## Results

### Participant characteristics

Table 1 outlines the demographic and infertility characteristics of the patients with infertility. The mean age and infertility duration of the sample were 32.97 ± 5.34 and 5.55 ± 4.07 years, respectively, and 53.8% were female. More than half (50.4%) of the participants had a university education, and 53.1% had at least one failure in previous infertility treatment.

**Table 1:** Demographic and clinical characteristics of the participants (*n* = 539)

	Mean ± SD or *n* (%)
Age (years)	32.97 ± 5.34
Sex	
Male	249 (46.2)
Female	290 (53.8)
Educational level	
Primary	92 (17.1)
Secondary	175 (32.5)
University	272 (50.4)
Duration of infertility (years)	5.55 ± 4.07
Cause of infertility	
Male factor	223 (41.4)
Female factor	95 (17.6)
Both	112 (20.8)
Unexplained	109 (20.2)
Failure of previous treatment	
No	253 (46.9)
Yes	286 (53.1)
History of abortion	
No	382 (70.9)
Yes	157 (29.1)

*SD* Standard deviation

### Descriptive statistics of PHQ-9 and PHQ-2

Table 2 presents item wording, means, and standard deviation for the PHQ-9. The item means ranged from 0.35 (for item 9 “Thoughts that you would be better off dead or of hurting yourself”) to 1.36 (for item 4 “Feeling tired or having little energy”). The mean PHQ-9 total score was 8.47 ± 6.17 (range, 0–27), and 208 patients (38.6%) had score of ≥10, indicating moderate to severe depression. The mean PHQ-2 total score was 2.42 ± 1.86 (range, 0–6), and 235 patients (43.6%) had score of ≥3, indicating depressive disorder.

**Table 2:** Items wording and descriptive statistics, and internal consistency of the PHQ-9 and PHQ-2

	Mean	SD	Corrected item total correlation	Alpha if item deleted	Cronbach’s Alpha
1 Little interest or pleasure in doing things	1.19	1.05	0.589	0.834	
2 Feeling down, depressed, or hopeless	1.22	1.01	0.698	0.822	
3 Trouble falling or staying asleep, or sleeping too much	1.12	1.10	0.561	0.837	
4 Feeling tired or having little energy	1.36	1.03	0.634	0.829	
5 Poor appetite or overeating	0.98	1.07	0.506	0.842	
6 Feeling bad about yourself – or that you are a failure	0.78	1.06	0.626	0.829	
7 Trouble concentrating on things	0.79	1.00	0.422	0.850	
8 Moving or speaking so slowly that other people could have noticed	0.68	0.99	0.597	0.833	
9 Thoughts that you would be better off dead or of hurting yourself	0.35	0.80	0.511	0.842	
PHQ-9 Total Score	8.47	6.17			0.851
PHQ-2 Total Score	2.42	1.86			0.767

*SD* Standard deviation

### Normative data

Table 3 presents the normative data for PHQ-9 and PHQ-2 transformed into percentile scores.

**Table 3:** Percentiles for PHQ-9 and PHQ-2 scores by gender

Percentiles	Total		Male		Female	
	PHQ-9	PHQ-2	PHQ-9	PHQ-2	PHQ-9	PHQ-2
1	0	0	0	0	0	0
5	0	0	0	0	1	0
10	1	0	0	0	3	0
20	3	1	2	0	4	1
25	3	1	2	0	5	2
30	4	1	3	0	5	2
40	6	2	4	1	7	2
50	7	2	5	1	9	3
60	9	3	7	2	11	3
70	12	3	9	3	13	4
75	13	4	10	3	15	4
80	14	4	12	4	16	5
90	17	5	15	5	19	5
95	21	6	18	6	22	6
99	24	6	24	6	25	6

*PHQ-9* Patient Health Questionnaire-9; PHQ-2: Patient Health Questionnaire-2

### Internal consistency

The internal consistency of the PHQ-9 was good, with Cronbach’s alpha of 0.851. As seen in Table 2, Cronbach’s alpha value did not increase if an item was deleted from the scale. The corrected item-total correlations ranged from 0.422 to 0.698 which were above the minimum level of 0.3. The inter-item correlations among the items were statistically significant, ranging from 0.200 (between Item 5 and Item 7) to 0.622 (between Item 1 and Item 2). Taking the brevity of the PHQ-2 into account, its internal consistency was also good (Cronbach’s alpha = 0.767).

### Convergent validity

Convergent validity of the PHQ-9 was demonstrated by the moderate to high correlations between PHQ-9 and four following relevant measures: WHO-5 (*r* = − 0.522, *P* < 0.001), HADS-D (*r* = 0.572, *P* < 0.001), HADS-A (*r* = 0.698, *P* < 0.001), and GAD-7 (*r* = 0.737, *P* < 0.001). Similar correlations were also obtained for PHQ-2 (Table 4). In addition, comparison indicated that the correlations for PHQ-9 were stronger than the correlations for PHQ-2. There was strong correlation between the PHQ-9 and PHQ-2 (*r* = 0.816, *P* < 0.001); however, due to the two identical items, this correlation needs to be interpreted cautiously.

**Table 4:** Correlations of the PHQ-9 and PQH-2 with measures of HADS, WHO-5, and GAD-7

	HADS		WHO-5	GAD-7
	HADS-A	HADS-D		
PHQ-9	0.698	0.572	−0.522	0.737
PHQ-2	0.573	0.491	−0.518	0.582

*PHQ-9* Patient Health Questionnaire-9, *PHQ-2* Patient Health Questionnaire-2, *HADS* Hospital Anxiety and Depression Scale, *WHO-5* WHO-5 Well-Being Index, *GAD-7* Generalized Anxiety Disorder-8

All correlations were significant at the 0.001 level

### Confirmatory factor analysis

**Fig. 1:**
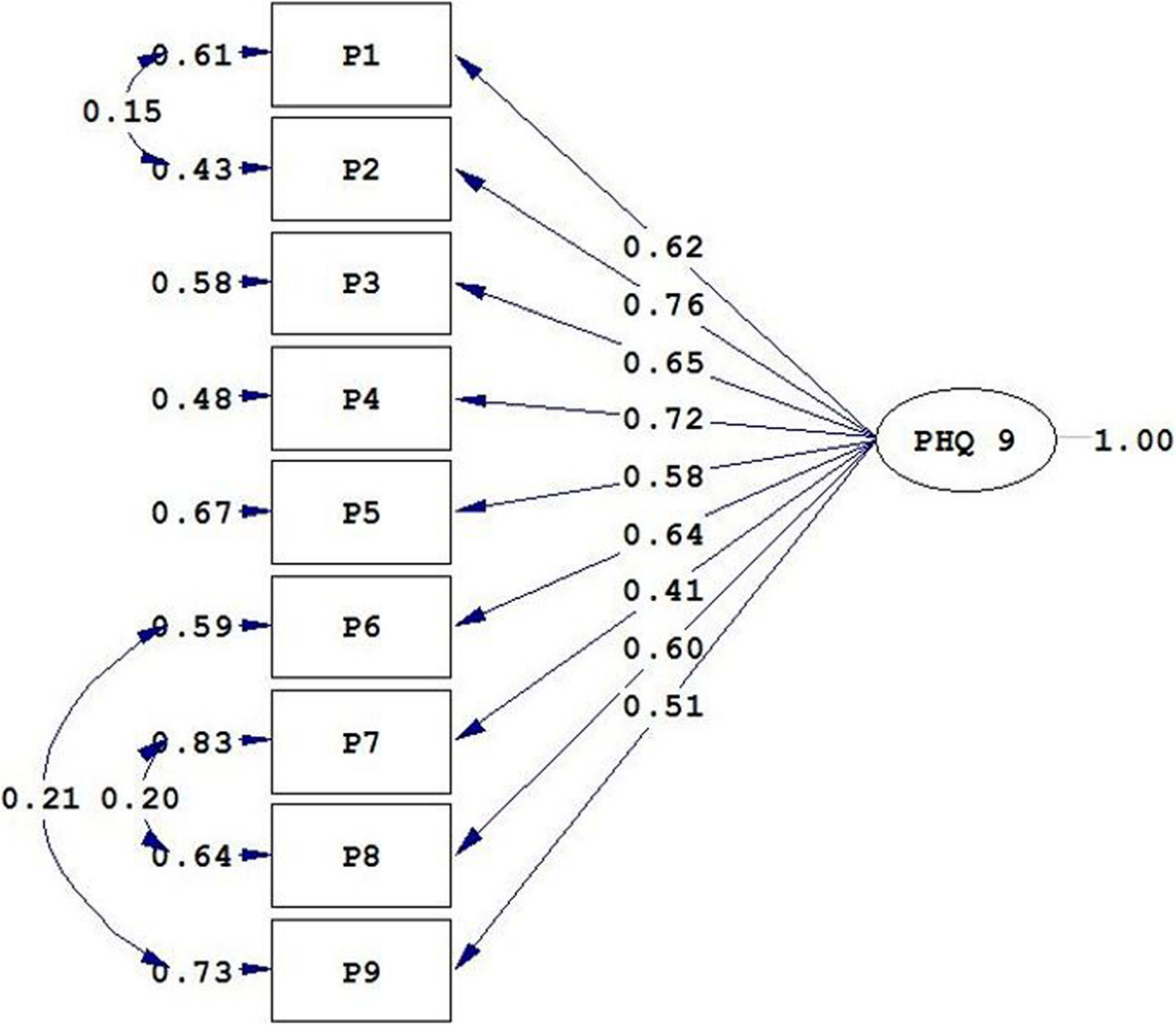
Unidimensional structure of the PHQ-9

To test the unidimensionality of the PHQ-9, the CFA was used. According to the goodness of fit indices, the fitness of the model was not good (χ^2^/df = 8.79; CFI = 0.94; RMSEA = 0.120 and SRMR = 0.059). Examination of the modification indices recommended allowing covariance between Item 1 and Item 2 as well as between Item 7 and Item 8, and Item 6 and Item 9 (Fig. 1). It means that there are high correlations between items. A better fit was obtained after allowing for these covariances (χ^2^/df = 4.29; CFI = 0.98; RMSEA = 0.078 and SRMR = 0.044). As shown in Fig. 1, all nine factor loadings were significant and in the expected direction.

### Relationship of the PHQ-9 and PHQ-2 with demographic characteristics

The relationships of the PHQ-9 and PHQ-2 with demographic/infertility characteristics of participants are presented in Table 5. There were significant but low positive correlations between duration of infertility and scores of PHQ-9 and PHQ-2 (*r* = 0.174, and 0.118, respectively). Women obtained higher scores on both PHQ-9 and PHQ-2 compared to men. Patients who had failure in previous treatment exhibited higher scores of PHQ-9 and PHQ-2 compared to patients undergoing first treatment. Other demographic and fertility variables were not correlated with either PHQ-9 or PHQ-2 scores.

**Table 5:** Relationship of PHQ-9 and PHQ-2 with demographic and infertility characteristics

	PHQ-2		PHQ-2	
	Mean (SD) or r^a^	P	Mean (SD) or r	P
Age (years)	0.025	0.560	−0.014	0.741
Duration of infertility (years)	0.174	< 0.001	0.118	0.006
Sex		< 0.001		< 0.001
Male	6.84 (5.67)		1.90 (1.83)	
Female	9.88 (6.25)		2.86 (1.77)	
Educational level		0.152		0.731
Primary	9.59 (6.46)		2.54 (1.91)	
Secondary	8.10 (6.18)		2.35 (1.83)	
University	8.33 (6.05)		2.41 (1.86)	
Cause of infertility		0.968		0.651
Male factor	8.38 (6.15)		2.42 (1.87)	
Female factor	8.49 (6.16)		2.21 (1.71)	
Both	8.38 (5.61)		2.51 (1.81)	
Unexplained	8.72 (6.83)		2.50 (2.02)	
Failure of previous treatment		0.009		0.022
No	7.74 (5.92)		2.22 (1.88)	
Yes	9.12 (6.33)		2.59 (1.83)	
History of abortion		0.057		0.157
No	8.15 (6.05)		2.34 (1.81)	
Yes	9.26 (6.42)		2.59 (1.95)	

*PHQ-9* Patient Health Questionnaire-9, *PHQ-2* Patient Health Questionnaire-2, *SD* Standard Deviation

^a^Pearson correlation coefficient

## Discussion

The aim of this study was to assess the reliability and validity of PHQ-9 and PHQ-2 among patients with infertility. The internal consistency of the PHQ-9 was good. Furthermore, the inter-item correlations as well as the corrected item-total correlations were also within acceptable range. Taking the brevity of the PHQ-2 into account, the internal consistency reliability of this version was also relatively good. These results are consistent with what was reported in previous studies in different populations [15–21]. Both PHQ-9 and PHQ-2 scores evidenced convergent validity by being correlated with other measures of depression (i.e. HADS-D and WHO-5) and anxiety (i.e. HADS-A and GAD-7) in expected ways. These findings are compatible with previous studies indicating that PHQ-9 (and PHQ-2) score is correlated with measures of depression, anxiety, well-being, mental health and quality of life [15–20].

The CFA results indicated that unidimensional structure of the PHQ-9 in patients with infertility had relatively adequate fit to the data. However, a better fit was obtained after allowing for covariance between some items. Most previous factor analysis studies reported on-factor structure for the PHQ-9 [22–26]. However, in some studies, a two-factor structure (somatic and affective factor) was supported by factor analysis [39–41].

In the present study the depression prevalence according to the PHQ-9 was 38.6%, which is considerably higher than what was reported in the general population [21, 42–44]. In a study conducted by Maroufizadeh et al. [9] among patients with infertility in Iran, the prevalence of depression using the HADS-D was 33.0%. In our study, the relationship of PHQ-9 and PHQ-2 scores with demographic/infertility variables was also examined. Consistent with previous studies [4, 8], women were more likely to report depression symptoms than men. This finding suggests that women were more affected by infertility problem than men in health and psychological status. Duration of infertility was positively correlated with both PHQ-9 and PHQ-2 scores, as patients with long infertility duration had more depression symptoms. This finding is in line with previous studies [4, 11, 45]. In keeping with one previous study [12], patients with at least one unsuccessful treatment had the higher level of depression compared to patients who undergo first treatment.

The PHQ-9 is an economical instrument that can be administered in only a few minutes and is easy to score. On the other hand, this scale evaluates each of the 9 DSM-IV criteria for major depressive disorder. The two-item version (i.e. PHQ-2) also provides a useful instrument when data must be collected over the phone or in internet-based research. In addition, as these scales are recommended by international guidelines [46], researchers should use the PHQ-9 (and PHQ-2) to screen patients for depressive symptoms in research and practice.

Our study had some limitations. First, the data was sampled from infertile people in a single-center setting and one must be cautious to generalize the results to other populations. Second, regarding some practical reasons, the test-retest reliability was not done among respondents. Third, to compare the sensitivity and specificity of PHQ-9 and PHQ-2 to a gold standard instrument, Structured Clinical Interview for DSM or another clinical interview is needed. Fourth, since this study is cross-sectional in nature, it is not possible to infer causality between study variables. Fifth, we did not collect the data on mental and somatic comorbidities.

## Conclusion

Both PHQ-9 and PHQ-2 are brief and easy to use measures of depressive symptoms with good psychometric properties that appears suitable for routine use in patients with infertility.

## Data Availability

The datasets of the present study are available from the corresponding author on reasonable request.

## Abbreviations

BDI-II: Beck Depression Inventory-II
CES-D: Center for Epidemiologic Studies Depression Scale
CFA: Confirmatory Factor Analysis
CFI: Comparative Fit Index
GAD-7: 7-item Generalized Anxiety Disorder
HADS: Hospital Anxiety and Depression Scale
PHQ-2: Patient Health Questionnaire-2
PHQ-9: Patient Health Questionnaire-9
RMSEA: Root Mean Square Error of Approximation
SD: Standard Deviation
SRMR: Standardized Root Mean Square Residual
WHO-5: WHO-5 Well-Being Index
